# Phytochemicals for Improving Aspects of Cognitive Function and Psychological State Potentially Relevant to Sports Performance

**DOI:** 10.1007/s40279-018-1007-0

**Published:** 2019-01-22

**Authors:** David O. Kennedy

**Affiliations:** Brain, Performance and Nutrition Research Centre, Northumbria University, Newcastle-upon-Tyne, NE1 8ST UK

## Abstract

Subjective alertness and optimal cognitive function, including in terms of attention, spatial/working memory and executive function, are intrinsic to peak performance in many sports. Consumption of a number of plant-derived ‘secondary metabolite’ phytochemicals can modulate these psychological parameters, although there is a paucity of evidence collected in a sporting context. The structural groups into which these phytochemicals fall—phenolics, terpenes and alkaloids—vary in terms of the ecological roles they play for the plant, their toxicity and the extent to which they exert direct effects on brain function. The phenolics, including polyphenols, play protective roles in the plant, and represent a natural, benign component of the human diet. Increased consumption has been shown to improve cardiovascular function and is associated with long-term brain health. However, whilst short-term supplementation with polyphenols has been shown to consistently modulate cerebral blood-flow parameters, evidence of direct effects on cognitive function and alertness/arousal is currently comparatively weak. Terpenes play both attractant and deterrent roles in the plant, and typically occur less frequently in the diet. Single doses of volatile monoterpenes derived from edible herbs such as sage (*Salvia officinalis/lavandulaefolia*) and peppermint (*Mentha piperita*), diterpene-rich *Ginkgo biloba* extracts and triterpene-containing extracts from plants such as ginseng (*Panax ginseng/quinquefolius*) and *Bacopa monnieri* have all been shown to enhance relevant aspects of cognitive function and alertness. The alkaloids play toxic defensive roles in the plant, including via interference with herbivore brain function. Whilst most alkaloids are inappropriate in a sporting context due to toxicity and legal status, evidence suggests that single doses of nicotine and caffeine may be able to enhance relevant aspects of cognitive function and/or alertness. However, their benefits may be confounded by habituation and withdrawal effects in the longer term. The efficacy of volatile terpenes, triterpene-rich extracts and products combining low doses of caffeine with other phytochemicals deserves more research attention.

## Key Points

**Table:** 

Evidence collected in normal healthy samples suggests that secondary metabolite phytochemicals from each of the main structural groups—phenolics (polyphenols), terpenes and alkaloids—may result in improvements to cognitive function and psychological state that could be relevant to sports performance.
A lack of research collected in the context of sporting performance limits the extent to which these results can inform practical recommendations.

## Cognitive Function Relevant to Sporting Performance

Optimal cognitive functioning is essential for peak sporting performance. Indeed, aspects of cognitive function enjoy a bi-directional relationship with sporting activity. Whilst the evidence is not entirely unequivocal [1, 2], some research suggests that athletes outperform non-athletes in terms of tasks measuring processing speed and attention [3], executive function [4], and spatial and working memory [5], but that different types of regular sporting activity with dissimilar cognitive requirements can be associated with corresponding differences in the performance of cognitive tasks measuring, for instance, attention task performance [3], executive function [4] and working memory capacity [6].

A common-sense case could clearly be made for the intrinsic contribution of efficient brain function, for instance in cognitive domains such as attention (which incorporates psychomotor-speed/reaction-times), spatial/working memory and executive function, to all forms of sporting performance. Clearly the comparative contribution of each domain would be dependent on the demands of differing sports [1, 3, 4, 6]. Similarly, modulation of a subjective psychological state, particularly increased alertness and decreased mental fatigue, will naturally have a knock-on effect on cognitive function, motivation and performance. All of these aspects of cognitive function and psychological state are amenable to modulation by the consumption of selected plant-derived ‘phytochemicals’.

Unfortunately, there is a complete absence of research assessing the psychoactive properties of most phytochemicals in a sporting context. There is also a lack of research investigating how and to what extent an improvement in any specific aspect of cognitive function or psychological state will result in improved sporting performance. Even for caffeine, which has benefitted from substantial relevant research, there is a paucity of research assessing the contribution that improved alertness and attention, as opposed to caffeine’s direct effects on physical performance, make to its beneficial effects on sporting performance (see Goldstein et al. [7]). As there are very few data collected in a sporting context, the following necessarily comprises a brief review of the evidence for potentially relevant cognitive/mood improvements drawn from the wider human controlled-trial literature. Whilst it seems reasonable to assume that the findings from this body of research would apply at least equally in a sporting context, the possibility that the increased physiological activation, psychological arousal or social interactions involved in some sports might have an interactive relationship with the psychological effects of phytochemicals has not been empirically investigated.

## Phytochemicals

As rooted, stationary autotrophs, each genus or species of plant has evolved the capacity to synthesise a unique mixture of ‘phytochemicals’. These ‘secondary metabolites’ play no role in the plant’s primary metabolism and have no macronutrient nutritional value. However, their synthesis increases the plant’s overall ability to overcome local challenges, by allowing the plant to interact with its environment. The roles here encompass general protection (e.g. antioxidant, ultra-violet light-absorbing and anti-proliferative properties); management of the plant’s relationship with pathogenic and symbiotic microorganisms above and below the soil; defence against local competitor plants; and management of the plant’s relationship with more complex organisms [8–10]. The latter includes both the deterrence of herbivory and attraction of pollinators and other symbiotes via colour and volatile chemical emissions. The primary relationships in these latter categories are with insects, the most abundant and prolific family of animals and the plant’s most intimate and co-evolved neighbour both in terms of herbivory and pollination. In this regard, it is relevant that, courtesy of a shared genetic heritage, insects and humans share functionally similar nervous systems, including in terms of all aspects of neurotransmission and cellular signal transduction mechanisms and molecules [8–10].

Most secondary metabolite phytochemicals can be classified into one of three structural groups: phenolics, terpenes and alkaloids. These groups play different ecological roles in the plant. Phenolics are present in all plant material where they play primarily protective roles in the face of environmental stressors. They provide colour, antioxidant and antimicrobial protection, particularly in the outer layers of leaves, fruits, etc. Terpenes play dual roles depending on the plant tissue and mode of delivery: the lower molecular weight volatile terpenes act as attractants for pollinators or symbiotes at low, airborne doses, but as toxic deterrents at the higher concentrations found in plant tissue and on the surface of leaves. More complex terpenes tend more towards toxic deterrence, including via interactions with the nervous and hormonal systems of potential herbivores. The last structural group, alkaloids, play almost exclusively toxic roles, again potentially via direct interactions with the nervous systems of herbivores [8–10].

These differing ecological roles correspond in many ways with the functional effects of these structural groups in human consumers. Phenolics, a ubiquitous component of the human diet, will tend towards exerting a global, beneficial effect on human physiological function, whereas both terpenes and alkaloids have evolved to exert multifarious direct, purposeful interactions with the nervous systems of symbiotic or herbivorous animals. This latter property can translate into an ability to modulate human brain function in the short term.

## Phenolics

The most intensively studied group of phytochemicals, the ‘phenolics’, can be broadly divided into simple phenolics, which incorporate one phenyl aromatic hydrocarbon ring within their structure and polyphenols, which incorporate two aromatic rings [11]. Each group can be further subdivided, with most polyphenols falling into the flavonoid sub-group, which can be further sub-divided into the chalcones, the flavanones and their derivatives the flavones, flavonols, isoflavones, flavanols and anthocyanins [12]. The flavanols comprise the most complex and varied sub-class of flavonoid structures, which range from single molecules (monomers), such as catechin and epicatechin, to chains of two (dimers) or more (oligomers/polymers) molecules, which are often referred to as proanthocyanidins or condensed tannins [13]. Unlike the small monomers (and some dimers), which can be absorbed in the upper intestine, the more complex structures first have to undergo metabolism by gut microbiota prior to absorption, and often ultimately appear in the circulation in the form of simple phenolics.

Polyphenols are present in most plant materials and are an unavoidable part of the human diet, with the greatest quantities being consumed in the form of alcoholic and non-alcoholic beverages such as wine and tea, fruit and fruit juices, and vegetables [14]. However, food diary studies suggest a wide variability in flavonoid consumption. For instance, the populations of the USA, Spain, Australia and France have been estimated to consume approximately 190 mg, 310 mg, 450 mg and 550 mg of flavonoids per day, respectively, with the largest part being taken in the form of flavanols and their oligomers and polymers [14–16]. Although receiving less attention, simple phenolics tend to be consumed at higher levels than polyphenols [17, 18].

Although polyphenols are typically described as antioxidants, the current consensus is that their effects are attributable to direct interactions within cellular signal transduction pathways [9, 19]. Functional effects here can include direct interactions with cellular receptors, but are more likely predicated on interactions within the protein-kinase and lipid-kinase signalling cascades [e.g. mitogen-activated protein kinase (MAPK) and phosphatidylinositide 3-kinase/protein kinase B (PI-3K/PKB)] that ferry signals received from outside of the cell, often by membrane or nuclear receptors, towards the cell’s nucleus. Targets also include cellular signal collection and processing mechanisms such as the ubiquitous energy-regulating target of rapamycin (TOR), which collects and rationalises information about external conditions, and internal energy availability and expenditure from multiple cellular signal transduction cascades [9, 20–23]. The summed and interacting activity in these signal transduction pathways dictate the cell’s response to environmental, stressor or internal energy-related information, for instance by modulating the activity of transcription factors [e.g. nuclear factor kappa-B (NF-kB) or cAMP response element-binding protein (CREB)]. This in turn leads to a wide range of cellular responses, including cell proliferation, apopotosis, the synthesis of growth factors and inflammatory molecules such as ‘induced’ nitric oxide synthase (iNOS), cytokines and cyclo-oxygenase-2 (COX-2). Overactivity or dysregulation within these signalling pathways is implicated in the pathogenesis of cardiovascular and neurodegenerative diseases and cancers [22, 24]. Interestingly, most of the mammalian cellular effects of polyphenols take place in the components of cellular signal-transduction cascades that are encoded by the ~ 3000 conserved genes that plants and humans share [9].

Of particular relevance to the brain, these signal transduction effects encompass, for instance: direct neurotransmitter receptor interactions; an increase in the synthesis of the neurotrophins that are integral to synaptic plasticity; and interactions with the ‘receptor tyrosine kinase’ membrane receptors that transduce neurotrophin signals, and their signalling cascades [25]. Interactions within kinase and TOR signalling pathways also modulate the synthesis of the vasodilatory molecule nitric oxide, leading to an increase in local cerebral blood flow, which in turn fosters angiogenesis/neurogenesis. These, and the more general cellular effects of phenolics, may contribute to both short-term benefits to brain function and neuroprotection/neuronal repair in the face of ageing and insults [21, 22, 26].

Whilst nitric oxide synthesis may partially underpin short-term cardiovascular benefits, polyphenols have also been shown to attenuate the inflammation and oxidative stress implicated in the pathogenesis of cardiovascular disease and tumorogenesis [27], and the neuronal damage associated with neurodegenerative diseases and the deterioration in cognitive function seen with ageing [28]. One further mechanism that is coming under increasing scrutiny is the role of dietary phenolics in the modulation of the gut microbial communities (which play a bi-directional role in cardiovascular and brain function)—a role that mirrors their management of nutrient-absorbing, symbiotic microbial populations in the plant’s root system [8, 9].

In line with these mechanistic effects, epidemiological evidence suggests that dietary phenolic consumption confers a wide range of health benefits. For example, individual cohort studies and multiple meta-analyses of the body of epidemiological research show that the consumption of polyphenols or polyphenol-rich foods is inversely related to all aspects of cardiovascular disease, including mortality [29–34]; protection against cerebrovascular diseases and neurological disorders, including dementia [35–42]; improved cognitive function in healthy middle age [43]; and reduced cognitive impairment and cognitive decline in elderly populations [36, 38, 44–46].

The cardiovascular benefits have also been confirmed in a large body of controlled intervention trials, principally involving polyphenols derived from cocoa, which are described briefly below. This having been said, the direct evidence of benefits related to physical performance or physiological parameters in recreational, competitive or elite athletes is currently insufficient to arrive at any conclusion as to the efficacy of polyphenol supplements in this regard [47].

The literature relating to several of the sources of polyphenols and individual compounds that have garnered significant amounts of relevant human research in non-sporting contexts are described below.

### Cocoa Flavanols

The largest body of human polyphenol research has involved the administration of cocoa-derived products, which are particularly rich in the flavanols catechin and epicatechin and their dimers/oligomers. Research here has benefitted from the availability of several standardised high-flavanol products. To date there is a lack of research assessing the effects of cocoa-flavanols on brain function in a sporting context [48], with the exception of a recent study that reported independent improvements in executive function following 30 min of moderate intensity cycling and a high cocoa-flavanol (536 mg flavanols) drink [49]. However, this study did not measure cognitive function during exercise or relate this to any aspect of performance.

Meta-analyses of the data from many dozens of intervention trials demonstrate a consistent beneficial effect of cocoa-flavanols on cardiovascular parameters, including inflammatory biomarkers related to atherosclerosis, insulin resistance, lipid profiles, blood-pressure and peripheral blood flow as measured by flow-mediated dilatation (FMD) [32, 50–52]. These meta-analyses suggest that these effects are achievable with an optimal dose of ~500 mg of flavanols per day [52] and within 2 h of consumption of the first dose [53]. It should be noted that many cocoa-derived products contain some carbohydrates, and almost all contain potentially bioactive levels of caffeine and other alkaloid methylxanthines. However, controlled trials typically match their comparator control interventions for these components.

In terms of brain function, increases in cerebral blood-flow (CBF) have been seen following single doses of cocoa extract containing ~ 500 mg [54] and 900 mg [55] of flavanols, using functional magnetic resonance imaging (fMRI) and trans-cranial Doppler (TCD), respectively. Cocoa extracts containing 170 mg [56] and 450 mg [57] of flavanols, administered sub-chronically for 5 days and 2 weeks, respectively, also increased CBF in healthy older and younger adults as assessed by fMRI and near-infrared spectroscopy (NIRS). Only one study has failed to report any CBF effects to date, in this case following both a single dose and 4 weeks’ administration of a low-caffeine cocoa extract containing 250 mg flavanols in comparison to a caffeine-free control [58].

In terms of psychological function following single doses, controlled crossover trials have demonstrated improvements on cognitively demanding tasks and reduced mental fatigue following the lower of two single doses of cocoa-flavanols (520 mg/994 mg) [59], and improved spatial memory, choice reaction time and visual sensitivity following flavanol-enriched dark chocolate (720 mg) [60]. One study also reported improved performance on a focussed attention task following brewed cocoa containing 500 mg of flavanols and 20 mg of caffeine versus a caffeine-free placebo [61], and one study reported no effects following cocoa drinks containing 250 mg and 500 mg of flavanols [62].

With regards chronic dosage, the best evidence of benefits comes from a pair of methodologically identical studies carried out in 90 healthy elderly participants [63] and 90 sufferers from age-related cognitive impairment [64], respectively. In both studies, participants received a low (control), medium (520 mg) or high (990 mg) flavanol drink for 4 weeks. In both studies the high-flavanol drink was associated with reduced insulin resistance, blood pressure and lipid peroxidation, and improved performance on two cognitive tasks that assessed attention and executive function. These findings were mirrored by those following the medium-flavanol treatment, with the exception that this dose did not improve the attention task. These findings are supported by a study also undertaken in 40 elderly participants in which supplementation with 500 mg of flavanols for 12 weeks resulted in improvements on a composite cognitive score derived from a battery of tasks. In this study, blood levels of brain-derived neurotrophic factor (BDNF), a protein involved in the survival, growth and differentiation of new neurons and synapses in the brain, were also increased in the flavanol condition [65].

Whilst these studies look promising, several studies involving the administration of 250–750 mg of flavanols for 4–6 weeks to middle-aged/older participants have failed to elicit any relevant improvements in cognitive function or psychological state [62, 66, 67]. It may be relevant that only one of these null studies reported matching the caffeine levels in their control treatments [62].

### Fruit Polyphenols

Fruit polyphenol interventions typically contain a complex mixture of simple phenolics and polyphenols. Comparator interventions in this research are typically matched for carbohydrate content if necessary. Whilst no studies have assessed the effects of fruit polyphenols on sporting performance, increases in CBF as assessed by arterial spin labelling fMRI have been seen in healthy adults, in comparison to a control, following a single dose of orange juice containing 70 mg of flavonoids [68] and after 12 weeks’ administration of a blueberry extract containing 387 mg of anthocyanidins [69]. A previous study also demonstrated increased brain activation (fMRI) and memory task performance, alongside increased plasma biomarkers of polyphenol absorption, in middle-aged and older participants following 4 weeks’ administration of pomegranate juice [70].

In terms of studies assessing psychological functioning, several crossover studies in healthy adults have reported subtle, treatment-related differences in cognitive function following single doses. For instance, two crossover studies involving the administration of anthocyanin-rich fruit juice containing 500 mg [71] and 140 mg [72] of anthocyanins reported benefits that were restricted to the performance of attention tasks, with the latter study also reporting improved subjective ratings of calmness. One study [73] found that orange juice (272 mg of flavanones) improved one of two psychomotor ‘finger tapping’ tasks and one attention task (out of nine tasks) and increased alertness. However, studies involving single doses of orange juice containing 70 mg of flavanones [68] and flavonoid-rich homogenised apple (360 mg of flavonoids) [74] found no significant differences compared with their control arms.

Two chronic dosage crossover studies have also been reported. In one [75], high (305 mg) versus low (37 mg) flavanone orange juice administered for 8 weeks to 37 older adults resulted in subtle improvements on one global measure of cognitive function performance. However, grape juice containing 770 mg of polyphenols consumed for 12 weeks by a small cohort of healthy working mothers did not result in any readily interpretable cognitive, mood or driving task benefits [76]. Two small studies also assessed the cognitive effects of blueberry drinks containing 143 mg [77] and 125/250 mg of anthocyanins [78] in children, with only the latter study demonstrating mild dose-related cognitive benefits.

### Single Molecule Polyphenols: Resveratrol, Curcumin and Quercetin

Resveratrol and curcumin are both non-flavonoid polyphenols (a stilbene and a curcuminoid, respectively) that are regularly consumed as single compounds. On the basis of preclinical and animal research it has been suggested that resveratrol has a number of properties relevant to the enhancement of physical or sporting performance, although this has not been investigated in humans to date [79]. This polyphenol has been shown to improve peripheral blood-flow, as assessed by FMD, in overweight/obese humans following a single dose and extended supplementation [80, 81]. Similarly, single doses of resveratrol (250 mg and 500 mg) have been shown to increase CBF in the frontal cortex during task performance in healthy young adults, as assessed by NIRS [82–84]. Single doses of 75 mg, 150 mg and 300 mg also increased CBF responses during hypercapnia in type II diabetics as assessed with TCD [85], with the lowest dose also increasing the haemodynamic response to cognitive task performance [86]. However, across these single-dose CBF studies only one reported concomitantly improved cognitive function, and this was restricted to two out of three doses of resveratrol [86].

In terms of chronic supplementation studies, 75 mg of resveratrol administered for 14 weeks to post-menopausal women resulted in an increased haemodynamic response to both hypercapnia and cognitive task performance as measured with TCD, alongside a general improvement in cognitive task performance [87]. Two placebo-controlled studies also assessed the effects of 26 weeks’ supplementation with resveratrol (200 mg/day) in 46 older adults [88] and 40 older adults with mild cognitive impairment [89]. Both studies demonstrated increased hippocampal connectivity as assessed by fMRI, but only the cognitively intact cohort saw cognitive improvements in the form of improved memory performance [88]. In contrast, Wightman et al. [82] demonstrated a lack of any interpretable cognitive effects of 28 days’ supplementation with 500 mg resveratrol, although subjective fatigue was reduced. A recent meta-analysis reported beneficial effects of resveratrol on cognitive function [90]. However, only three studies out of the ten that were included in the analysis reported benefits, and two of these three studies administered resveratrol alongside other polyphenols (red wine and quercetin, respectively).

Meta-analyses of the results of multiple human trials suggest that curcumin, the principal polyphenol found in turmeric, can improve serum lipid levels [91], gluco-regulation [92] and inflammatory cytokine levels [93], and that it may be effective in treating depression [94]. Recent studies have suggested protective anti-inflammatory properties in the face of exercise-induced muscle damage [95] and preservation of gastrointestinal barrier damage during heat stress exercise [96]. Interestingly, a recent study demonstrated that 8 weeks’ supplementation with curcumin in the absence of exercise resulted in a similar improvement in endothelial function to a programme of aerobic exercise alone [97].

There is less evidence with regard cognitive function, and no studies that have been conducted in a sporting context. In one study in older adults, both a single dose and 4 weeks’ administration of 400 mg of curcumin led to modestly improved cognitive function in terms of attention and working memory, with additional improvements in fatigue or alertness either before or after the day’s treatment after 4 weeks [98]. In the only other study involving healthy humans [99], there were no interpretable beneficial cognitive or mood effects of 12 months’ administration of 1500 mg of curcumin.

Evidence suggests that chronic consumption of the flavonoid quercetin may have a small beneficial effect on endurance exercise (at around 1 g per day) in humans [100], although the evidence is somewhat equivocal [47]. However, whilst there is some evidence of improved cognitive functioning following this single-molecule polyphenol in rodents, only one study has assessed psychological functioning in humans, with no effect of 12 weeks’ supplementation seen in a large sample of young, middle-aged and older adults [101].

### Other Polyphenols

Several other polyphenol groups have attracted some research attention in this area. A review of studies assessing the cognitive effects of estrogenic soy-isoflavones [102] described equivocal evidence across 11 studies, mainly in samples of post-menopausal women. Similarly, whilst a number of studies have assessed the effects of chronic supplementation with proanthocyanidin-rich pine bark extracts, the evidence of benefits in psychological functioning, to date, is less than clear [103–106].

### Conclusion

The epidemiological and meta-analysis evidence that suggests wide-ranging cardiovascular benefits associated with consuming polyphenols is credible and consistent. However, the case for benefits to cognitive performance or psychological states relevant to sport performance is less persuasive. There is clear evidence that polyphenol-rich products consistently increase CBF or modulate brain activity after single doses [54, 55, 68, 83–85, 107] and longer periods of supplementation [56, 57, 69, 70, 88, 108, 109]. However, whilst most of these CBF studies included an assessment of cognitive task performance or mood, only a very small minority [70, 86, 88] reported any significant improvements in these domains in comparison to placebo. In contrast, slightly more than half of the above studies that focussed solely on cognitive function and psychological state reported interpretable benefits in comparison to placebo. However, the benefits in several of these papers could best be described as subtle. This does suggest the potential for a publication bias within the literature in this area. Either way, the current evidence base suggests significant physiological health benefits for the consumption of polyphenols at levels that can be achieved in the diet, but the evidence with regard to the potential for phenolic supplements to improve aspects of cognitive function or psychological state relevant to sport performance in the short term is currently equivocal. Further research into the medium-/long-term effects of supplementation with phenolics is warranted.

## Terpenes

The terpenes comprise a large, structurally diverse family of 35,000 + compounds that are composed of units of the volatile, organic, 5-carbon compound isoprene, and classified according to the number of isoprene units they contain. The lower molecular weight volatile monoterpenes and sesquiterpenes incorporate two and three isoprene units, respectively, whereas diterpenes, triterpenes and tetraterpenes incorporate four, six and eight units, respectively [11]. All plants synthesise terpenes, both as primary metabolites and as the principle volatile components of the gaseous emissions, such as floral bouquets, that attract pollinators and symbiotes. However, only a minority of plant clades have evolved the specific use of terpenes in extensive ecological roles, such as defence against herbivory [8].

### Monoterpenes/Sesquiterpenes

A number of plant families rely on volatile terpenes not only as airborne attractants, but also as defensive agents [8, 110]. As attractants, plants synthesise and release comparatively low concentrations of mono- and sesquiterpenes. Many of these volatile terpenes are also produced endogenously by insects as intra-species (pheromone) and inter-species (allomone) communication chemicals [111]. When employed in a defensive role, the same volatile terpenes are typically manufactured and stored at high concentrations in cells external to the plant’s vascular system in order to avoid auto-toxicity. For instance, they can be synthesised in tiny stalk cells, called glandular trichomes, on the insect-favoured underside of the leaf, and stored in a balloon-like bladder on top of the stalk [112]. When a herbivore visits the plant the delicate bladders are broken open, delivering a toxic dose of sticky, terpene-rich oil onto the visitor. The key difference between attraction and toxicity is therefore the dose and mode of administration of the volatile terpenes. Indeed, the lower, airborne doses of volatile terpenes that are associated with attraction will not negatively affect insect neurotransmission and behaviour, and may have a beneficial effect, as befits their roles as insect pheromone/allomone chemicals. The higher, defensive doses will, on the other hand, interfere with the same aspects of neurotransmission [8].

The dual use of volatile terpenes in ecological roles is particularly prevalent within the large *Lamiaceae* family of plants. The *Nepetoideae* sub-family represents a particularly abundant source of monoterpene-rich culinary herbs and essential oils, which are distilled oils composed of the plant material’s volatile terpenes. In line with this, essential oils contained in the trichomes of plants from across this taxon generally exhibit dose-dependent insecticidal properties against adult insects, principally via interactions with the insect nervous system [113], and they kill and inhibit the growth of larvae from a number of species [114, 115].

Most of the psychoactive members of *Nepetoideae*, including rosemary, lemon balm, sage and peppermint, belong to the *Mentheae* tribe of plants. Essential oils from this group typically contain mono- and sesquiterpenes such as 1,8-cineole, α-pinene camphor, geraniol, geranial, borneol, camphene and β-caryophyllene [116, 117], with the differing aromas of family members being due to the differing concentrations of these. So, for instance, rosemary essential oil typically comprises 60% 1,8-cineole whereas peppermint primarily expresses menthol and menthone [118]. These plants also express comparatively low concentrations of diterpenes and triterpenes, but reasonably high levels of phenolics, including, in all cases, rosmarinic acid and its derivatives, alongside other phenolic acids and polyphenols [119–124].

Extracts from this family of plants share common (but variable) mechanisms of action relevant to the brain, for instance, inhibiting acetylcholinesterase and binding allosterically to gamma-aminobutyric acid_A_ (GABA_A_), nicotinic and muscarinic receptors [8]. For some of these mechanisms of action their overall potency depends on synergistic interactions between their monoterpene constituents [125–127]. These mechanistic effects do mean that some members of this plant group would be unlikely to have beneficial effects on cognitive function in a sporting context. The obvious examples here are *Melissa officinalis* (lemon balm) extracts, which exert GABA_A_ [128] and nicotinic/muscarinic receptor binding and cholinesterase inhibitory properties that depend on the nature of the extract [126, 129–131]. Research in healthy adults shows that lemon balm extracts can improve aspects of cognitive function [129, 130, 132], but that they can equally result in decreased alertness, increased fatigue and some cognitive decrements [130–132], depending both on the dose and the nature of the extract.

There is a lack of research into the effects of volatile terpenes in a sporting context [48]. Only the *Mentheae* that have resulted in consistent, potentially relevant psychological benefits will be considered below.

Interestingly, volatile terpenes, which are small lipophilic molecules, have been shown to have rapid bioavailability following passive inhalation and pulmonary absorption of vapour [133, 134]. It is also likely that purposeful intranasal inhalation of concentrated volatile terpenes confers a number of further brain bioavailability advantages, with additional absorption via the nasal cavity’s olfactory and epithelium mucosa [135] and potential intraneural and perineural transport along the trigeminal nerve and olfactory tract to a number of brain regions, including the piriform and entorhinal cortices, amygdala and hypothalamus [135]. Whilst passive inhalation has been investigated (e.g. Moss et al. [136, 137]—see section 4.1.2) the advantage of purposeful nasal inhalation in terms of brain function has not yet been investigated.

#### Sage (*Salvia officinalis*/*lavandulaefolia*)

The psychoactive effects of consuming single doses of cholinesterase inhibiting sage extracts have been assessed in a series of double-blind, placebo-controlled, crossover trials in healthy humans. The two lowest of four single doses (167/333/666/1333 mg) of an ethanolic extract of *S. officinalis* containing a full range of phytochemicals improved memory task performance in healthy elderly adults over the 6 h following consumption, with the lowest dose also improving attention task performance [138]. Similarly, both 300 mg and 600 mg of encapsulated dried sage improved mood in young adults, with the lower dose reducing anxiety and the higher dose improving subjective ratings of alertness and calmness [139].

Several studies have assessed the effects of essential oils composed solely of the volatile terpenes present in plant material in young adults. In the first of these the consumption of single doses of 50 µl and 100 µl of encapsulated *S. lavandulaefolia* essential oil [140] improved memory task performance over 2.5 h post dose. These effects were replicated following single doses of 25 µl and 50 µl of the same essential oil, along with improved performance of a working memory/executive function task and improved levels of subjective alertness, calmness and contentment [141]. Most recently, the psychoactive properties of cholinesterase inhibiting *S. lavandulaefolia* essential oil were confirmed in healthy young adults who consumed single doses of 50 µl oil composed exclusively of monoterpenes and with a high concentration of 1,8-cineol. Within the first 4 h, memory and attention task performance were improved alongside increased alertness and reduced mental fatigue during extended performance of difficult tasks [142]. One parallel-groups study has also assessed the cognitive and mood effects of *S. officinalis* and *S. lavandulaefolia* essential oil vapour versus a no-vapour control condition [143]. In these ‘aromatherapy’ studies, participants are exposed to vapour in a small enclosed room, with the volatile terpenes absorbed through the lungs and mucosa. In this instance, both sage vapours improved mood, but only *S. officinalis* improved memory performance in healthy young adults.

#### Rosemary (*Rosmarinus officinalis*)

Whilst the analgesic and antispasmodic effects of rosemary extracts may be due to GABA, opioid, muscarinic or serotonin (5-HT) receptor interactions [144–146], the psychoactive properties of rosemary may relate to cholinergic receptor binding and cholinesterase inhibitory properties [147–149].

A handful of studies have assessed the effects of rosemary on human brain function. In the earliest study, exposure to vaporised rosemary essential oil increased alertness and led to more accurate but slower memory task performance, in comparison to a control condition, in 140 young healthy participants [136]. This study was followed by two studies involving 20 and 64 participants in which plasma levels of 1,8-cineole were seen to increase alongside increased exposure to essential oil vapour, confirming the bioavailability of the monoterpenes via inhalation. The changes in performance of attention, working memory/executive function tasks [137] and a prospective memory task [150] also correlated with plasma levels of 1,8-cineole post administration.

Several randomised, double-blind studies have also assessed the effects of oral consumption of rosemary. In the first crossover study, 28 elderly adults consumed single oral doses of 750/1500/3000/6000 mg of powdered rosemary leaf on separate occasions. Assessments across the next 6 h showed improvements in alertness and the speed of performing memory tasks following the lowest dose, but significant decrements on the same measures following the highest dose, with mixed results for the intermediate doses [122]. Subsequently, a single-dose crossover study in 40 young adults demonstrated no effects of 1.7 g of dried rosemary leaf on a single sustained attention task and subjective ratings of psychological state [151]. One study has also assessed the effects of longer term consumption (4 weeks) of 500 mg of dried rosemary in 68 healthy young adults and found that the participants’ subjective ratings of their prospective and retrospective memory performance, anxiety and depression improved when taking rosemary. In this instance no objective measures of cognitive function were taken [152].

#### Peppermint (*Mentha piperita*)

Peppermint typically expresses high levels of the monoterpenes menthol and menthone [153, 154]. In vitro, essential oils and/or menthol alone have cholinesterase inhibitory properties [153, 155, 156] and interact with 5-HT_3_ [157], GABA_A_ [158–160] and glycine [158] receptors.

In one of two single-blind studies, peppermint tea increased alertness and improved accuracy and speed of memory task performance 20 min after consumption, in comparison to a hot water control, in 180 young adults [161]. In the second, the only study to date involving physical performance, pure peppermint essential oil dripped onto the tongue improved auditory and visual reaction times 5 min and 1 h after administration and improved lung function and physical performance (grip force, standing vertical and long jump) in comparison to a water control in 30 participants [162]. In the only double-blind, placebo-controlled study to date [118], 24 participants received 50 µl and 100 µl of encapsulated peppermint essential oil and placebo on separate occasions. In this instance, the essential oil had been selected on the basis of its in vitro ability to inhibit cholinesterase and bind to nicotinic and GABA_A_ receptors, with increased cellular activity confirmed in a neuronal cell line. Consumption of the highest dose of essential oil resulted in improved performance of a focussed attention task at 1 and 3 h post dose, and improved serial subtraction task performance and reduced mental fatigue at 3 h post dose. These effects had attenuated by 6 h post dose.

### Diterpenes

Most psychoactive diterpenes have an assumed provenance as insecticidal feeding deterrents. Examples include tanshinones from *Salvia miltiorrhiza*, which exert GABAergic and cholinesterase inhibitory properties, picrotoxin from *Anamirta cocculus,* which is a potent inhibitor at GABA_A_ receptors, and the hallucinogenic κ-opioid receptor agonist salvinorin A from *Salvia divinorum* [8].

#### *Ginkgo biloba*

The only contenders within this structural group with regard to evidence of relevant improvements in human cognitive function are the diterpene ginkgolides and their bilobalide derivatives from *Ginkgo biloba*. There is, however, a lack of evidence collected in a sporting context [48]. Interestingly, ginkgolides have potent insecticidal properties via GABA receptor inhibition, but mammals are spared this functional effect by a tiny, single amino-acid divergence in the morphology of our genetically conserved GABA receptor [163]. Ginkgolides and bilobalide typically make up 6% of standardised *G. biloba* extracts.

The vast bulk of the large research effort surrounding *G. biloba* extracts has focussed on dementia. Meta-analyses of the data from the many trials in this area suggest that *G. biloba* extracts exert promising effects with regard to cognitive function and behaviour in this patient group [164–168], although the evidence is not altogether unequivocal [169–171].

In healthy populations, standardised *G. biloba* extracts have been shown to increase CBF in brain-imaging studies following 4 weeks’ [172] and 8 months’ [173] supplementation. A number of randomized control trials have also demonstrated cognitive enhancement, including in terms of attention task performance, in young adults following single doses of *G. biloba* extract [174–179], and in both younger [180] and older [181–183] ‘cognitively intact’ populations administered *G. biloba* for 7 days or longer. More recent studies employing larger samples, and focussing on healthy middle-aged participants, have shown improved memory performance after 6 weeks’ supplementation in 188 participants [184], and improved attention, memory and subjective ratings of physical health in 300 participants administered *G. biloba* for 12 weeks [185].

### Triterpenes

Triterpenes play diverse ecological roles for the synthesising plant, including pronounced anti-feedant/anti-herbivore and soil-micro-organism management roles. Many of these functions are potentially related to the triterpene chemical structure, which is also the favoured structure for hormonal communications across phyla. Examples include mammalian sex hormones (estrogen/testosterone) and glucocorticoids (cortisol), invertebrate hormones, insect ecdysteroids and plant brassinosteroids. These structural similarities allow plant triterpenes to interfere with ecdysteroid function and therefore the life cycle of herbivorous insects [8], and potentially disrupt the endocrine function of invertebrates [186]. They also interact with pathogenic and symbiotic soil microorganisms via bacterial/fungal ‘estrogen-like’ receptors [8].

Many triterpene-containing herbal extracts are traditionally classified as ‘adaptogens’, a term that denotes that they primarily function by protecting the consuming organism from the negative impact of physical, biological, chemical and psychological stress. Typically they are theorised to do this by fostering homeostasis in the face of stressors by modulating the functioning of the hypothalamic–pituitary–adrenal (HPA) axis [187]. In this regard, a number of these triterpenes have been shown to interact with multiple mammalian steroid hormone receptors [188]. As an example, ginsenosides and their metabolites exert measurable in vivo effects on many aspects of HPA axis function and interact directly at multiple steroid hormone receptors including ubiquitous estrogen and glucocorticoid receptors [188]. Similarly, plant-derived phytoecdysteroids, which mimic the structure of insect hormones, are available as body-building supplements, and they have their effects on mammalian muscle mass via interactions with mammalian nuclear hormone receptors [189]. The global effects of interactions with the ubiquitous glucocorticoid receptor alone could encompass many of the diverse physiological effects of this class of phytochemicals, including effects on neurotransmission.

#### Ginseng (the *Panax* genus)

The active components of ginseng (members of the *Panax* genus) extracts comprise 40 or more triterpene ‘ginsenosides’ [190]. Many of the physiological effects of ginseng can be accommodated by modulation of nitric oxide synthesis [190, 191]. For instance, *Panax ginseng* has been shown to have greater utility in treating ischemic heart disease than nitrates [192], and is an effective treatment for erectile dysfunction [193]. In contrast, whilst the ergogenic effects of ginseng have been investigated in numerous studies, the evidence to date is equivocal, potentially due to methodological issues within this literature [194, 195].

Nitric oxide synthesis may be one consequence of either the genomic or non-genomic effects of modulating the glucocorticoid system, as described in Sect. 4.3. These nuclear receptor interactions may also underpin the multifarious effects relevant to brain function, which include indirect modulation of GABA_A_, serotonin, nicotinic and glutamate *N*-methyl-d-aspartic acid (NMDA) receptor function, and modulation of cellular signal transduction [188, 196].

In terms of human brain function, only one double-blind, parallel-groups study assessed psychological functioning in a sporting context, demonstrating improved reaction times on a psychomotor task both before and during graded cycle ergometer exercise in a small sample of 15 athletes [197].

In a non-sporting context a number of randomised, controlled, balanced-crossover, single-dose (200–400 mg) trials have demonstrated consistent improvements in the accuracy of memory tasks [198–200] and the speed of attention task performance [200, 201]. Single doses have also decreased the latency of cerebro-electrical evoked potentials as measured by electroencephalography (EEG) [202], and improved the performance of difficult working memory/executive function tasks and concomitantly reduced mental fatigue [203, 204]. A recent study [205] also showed that 7 days’ supplementation increased ‘calmness’, following both doses investigated (200/400 mg), and improved performance of the ‘3-back’ working memory task following the higher dose, but with slower performance following the lower dose. A subsequent study assessing the effects of 8 weeks’ administration of a Korean red ginseng extract also demonstrated improved working memory performance and modulation of ratings of ‘calmness’ [206]. A single study has also extended these findings to single doses of a standardised *Panax quinquefolius* extract, with demonstrations of improved working memory performance and dose-dependent increases in speed of performance [207].

#### *Bacopa monnieri*

The active constituents of *Bacopa monnieri* extracts are triterpene bacosides or bacopasides that are structurally similar to the ginsenosides [208, 209]. Whilst the exact mechanisms by which *Bacopa* modulates brain function remain unclear, animal research suggests interactions with the acetylcholine, opioid and GABA neurotransmitter systems [210–214] and the HPA axis [215].

In humans, whilst some research has assessed the acute cognitive and mood effects of bacopa [216, 217], none of this has taken place in a sporting context. A recent meta-analysis of the data from nine chronic dosage (> 12 weeks) randomised, controlled trials, incorporating 518 participants, found that supplementation with ~ 300 mg/day of bacopa extract (containing 50% triterpene bacosides) improved attention-task performance and speed of processing [218]. One of the included studies also demonstrated a decreased latency of evoked potentials to stimuli (i.e. faster cerebro-electrical processing) using EEG [219].

#### Other Triterpenes

Several other triterpene-rich herbal extracts have exhibited potentially relevant effects in humans. A meta-analysis of data from 11 studies in which *Centella asiatica* (Gotu kola) extracts, which contain triterpene asiaticosides, were administered for 60 days or more, either alone or in combination, demonstrated no effects on cognitive function, but significant increases in subjective alertness and calmness [220]. Similarly, an *Avena sativa* (wild green oat) extract containing triterpene avenacins has also been shown to improve peripheral and cerebral vasodilation in healthy participants [221] after 12 weeks’ supplementation with 1500 mg. A single dose (800 mg) of the same extract also increased the global speed of performing computerised tasks, and improved working memory and executive function task performance, in the 6 h after consumption. In this instance a higher dose (1600 mg) was less effective [222]. Single, placebo-controlled studies also demonstrated that *Withania somnifera* extract, which contains triterpene withanolides, administered for 60 days could improve attention, executive function and memory [223], and that *Rhodiola rosea* extract, which contains salidrosides, could increase the speed and accuracy of two attention tasks [224].

### Conclusion

Terpene phytochemicals have a number of specific brain function effects predicated on their dual ecological roles as insect attractants and/or deterrents. Monoterpenes derived from a number of edible and well tolerated herbs, particularly terpene-rich sage, rosemary and peppermint, have been shown to exert a number of potentially beneficial effects on human cognitive function and alertness/fatigue that would be relevant to sporting performance. Amongst the more complex non-volatile terpenes, diterpene-rich *G. biloba* extracts and several triterpene containing extracts also show some promise. In particular, single doses of standardised ginseng (*P. ginseng/quinquefolius*) extract and chronic supplementation with *B. monnieri* extracts have been shown to consistently improve cognitive function across several domains relevant to sport. However, there is a paucity of research assessing either the chronic effects of ginseng or the acute effects of *B. monnieri,* therefore no conclusion can be reached with regards these dosing regimens.

## Alkaloids

Alkaloids are a structurally diverse group of low molecular weight compounds that contain one or more nitrogen atoms, typically as part of an amine group. They are synthesised by approximately 20% of plant species, almost all of which are flowering plants. They play exclusively defensive ecological roles, most notably by protecting plants against herbivorous insects and invertebrates via toxicity. In this respect, interference with all aspects of neurotransmission is one of their hallmark functions. Because of the close similarities between insect, invertebrate and mammalian nervous systems, plant-derived alkaloids (sometimes slightly modified) provide us with numerous psychoactive compounds, including medicines, deliriants and most of our social and illicit drugs: nicotine, caffeine, cocaine, opiates, amphetamine and its many derivatives, and hallucinogens such as mescaline, psilocin and lysergic acid diethylamide (LSD). Given their toxicity, legal status and psychopharmacological properties, few alkaloids would confer any potential benefits in a sporting context, potentially with the exception of the two most commonly consumed psychoactive compounds: nicotine and caffeine.

### Nicotine

The brain function effects of nicotine are predicated on its excitatory binding to acetylcholine nicotinic receptors. The subsequent modulation of glutamatergic and GABAergic function leads to the release of other neurotransmitters, with dopamine driving nicotine’s addictive properties, GABA and β-endorphins mediating anxiolytic properties, noradrenaline contributing to the arousing effects, 5-HT driving mood effects, and 5-HT, dopamine and noradrenaline all serving to reduce appetite [225–227].

Regular nicotine use will create addiction and habituation, whereby nicotinic receptor populations and sensitivity change over time, leading to symptoms of withdrawal in nicotine’s absence. Much of the human literature is therefore confounded by conducting research in smokers who had abstained from smoking, making the interpretation of any net effects of nicotine difficult to disentangle from alleviation of decrements due to being in a state of a withdrawal. However, whilst there is plentiful evidence of nicotine exerting cardiovascular effects potentially relevant to improved physical performance in nicotine-naïve consumers [228], this evidence is not matched by any persuasive evidence of actual performance improvements [228, 229]. The story with regard to brain function in a non-sporting context is better. A comprehensive meta-analysis included the data from 50 methodologically adequate studies that had assessed the effects of nicotine administered via various methods in non-deprived smokers, minimally deprived smokers or non-smokers. The authors concluded that, irrespective of nicotine withdrawal, nicotine led to consistent improvements in cognitive performance in a number of domains, including fine motor tasks, the speed of response on attention and working memory tasks, and the accuracy of attention and short-term memory tasks [230].

### Methylxanthines*—*Caffeine

In plants, caffeine is typically synthesised as the most prominent of a group of structurally related methylxanthines. However, controlled trials suggest that non-caffeine methylxanthines such as theobromine and theacrine, which is present in some tea plants, have inconsistent or negative effects on mood, and no interpretable beneficial effects on cognitive function [231–236]. Caffeine, on the other hand, has consistent central nervous system effects, rapid bioavailability, with a half-life of 3–5 h, and the propensity to rapidly cross the blood-brain barrier [237]. Caffeine’s central nervous system effects at normal doses are predicated on its ability to inhibit the action of the inhibitory neuromodulator adenosine at adenosine A_1_ and A_2_ receptors distributed throughout the vasculature and neuronal tissue. The resultant release of inhibition leads to cerebral vasoconstriction and increased neuronal activity, with a net excitatory effect [238, 239]. Caffeine has well-established ergogenic properties and has been shown to enhance performance of both endurance exercise and high-intensity and intermittent exercise, for instance as associated with team sports. These benefits are attributed primarily to its effects on the central nervous system at moderate/high doses (~ 3–6 mg/kg or 225–450 mg for a 75-kg individual) [7]. Lower doses of caffeine, in the range from 32 mg to 300 mg, have been shown to enhance brain function, with effects plateauing or attenuating at higher doses [237]. However, caffeine’s effects on psychological function are limited to increased subjective arousal/alertness, and improved performance of psychomotor tasks and ‘simple’ attention, focussed attention and vigilance type tasks. Caffeine has inconsistent effects on working memory and executive function tasks and no interpretable effect on memory function [237, 238, 240]. As with nicotine, the use of caffeine in a sporting context is complicated by habituation and withdrawal.

#### Caffeine Interactions

Whilst it has been suggested that anhydrous caffeine may be more effective in terms of ergonenic benefits than coffee [7], a recent synthesis of the literature described good evidence that caffeine consumed in the form of coffee is also effective for improving performance during endurance exercise [241]. Evidence also suggests that caffeine may participate in additive or synergistic relationships when co-consumed with other bioactive compounds. For instance, the brain function effects of caffeine are differentially modulated, or vice versa, by the co-consumption of other phytochemicals [238], and food components such as amino acids [242–244], glucose [245, 246], choline[247], taurine [248, 249] and micronutrients [250, 251]. Co-consumption of comparatively low doses of caffeine has also been shown to increase the bioavailability of phenolic compounds [252, 253]. A recent study also showed that co-administration of caffeine/methylxanthines alongside cocoa-flavanols increased plasma flavanol metabolites and led to greater cardiovascular effects than cocoa-flavanols alone. Methylxanthines by themselves had no effect on any parameter [254]. This does raise the question of the potential synergistic contribution of caffeine to the effects of the cocoa-flavanol interventions employed in the numerous controlled trials that provide the bulk of the polyphenol literature (see Sect. 3.1). The interventions here typically contain 15–40 mg of caffeine, with a methylxanthine-matched control treatment, but no flavanol-only comparator.

#### *Caffeine*—*Guaraná* (*Paullinia cupana*)

One example of potential caffeine/phytochemical interactions is provided by guaraná (*Paullinia cupana*). The effects of guaraná seed extract are typically attributed to the presence of caffeine (2.5–5% dry weight). However, extracts also contain significant levels of polyphenols and triterpene saponins [255]. Controlled single-dose trials have shown that guaraná extract (75 mg) can improve attention, executive function and working memory tasks [200], and engender dose-related (37.5 mg, 75 mg, 150 mg and 300 mg guaraná) increases in alertness, ‘contentedness’ and memory task performance. However, most of these effects have been seen following doses of guaraná that contain non-psychoactive levels of caffeine (4.5 mg/9 mg) [255]. Single doses of multivitamin/minerals with added guaraná (220 mg including 40 mg of caffeine) have been shown to improve the performance of difficult attention and working memory tasks, alongside improvements in alertness or contentedness [250, 256], with mood benefits seen both before and after 30 min of treadmill running at 60% of their maximal oxygen consumption (*V*O_2max_) [257]. A study that compared a multivitamin/mineral plus guaraná to both placebo and its caffeine content found that guaraná improved executive function/attention task more than its caffeine content alone [258].

#### Conclusion

Courtesy of their ecological roles, which include the disruption of neurotransmission in herbivores, alkaloids have the most profound brain function effects amongst the phytochemical groups. At the low doses usually consumed by humans, both nicotine and caffeine are capable of engendering benefits to psychological functioning that could translate into performance benefits in a sporting context. However, these alkaloids are toxic at high doses, and they engender habituation via modulation of receptor populations and sensitivity, which could lead to symptoms of withdrawal, including potential performance decrements, in their absence. Nicotine is also highly addictive and therefore best avoided. The cognitive effects of these phytochemicals in a sporting context may therefore be most marked in those who either do not use these alkaloids regularly (for instance during training), or, particularly in the case of caffeine, when habitual consumption is low in comparison to the intended performance-enhancing dose. That having been said, a growing literature suggests that the co-consumption of comparatively low doses of caffeine alongside other bioactive compounds, for instance in the form of guaraná, may lead to additive or synergistic effects on cognitive performance and alertness.

## Conclusion

A number of phytochemicals have cognitive and alertness/arousal effects that may be relevant to improved sporting performance. Broadly, these effects can be related to the ecological roles of the phytochemical groups. Phenolics and their polyphenol subgroup, which currently attract the most research attention, are intrinsic to good health and cardiovascular function as part of the everyday diet. However, the evidence of additional benefits to cognitive function and mood following short-term supplementation is currently weak. This profile of functional effects is in keeping with their plant roles as general protectants. On the other hand, the terpenes and alkaloids discussed above, which are not a natural, unavoidable component of the diet, serve ecological roles that include interactions with the central nervous systems of symbiotes and/or herbivores. Terpenes tend to be overlooked, but the evidence of beneficial cognitive and mood effects is promising for several edible, monoterpene-expressing herbs such as sage, rosemary and peppermint; diterpene-rich *G. biloba*; and several triterpene-rich extracts, including ginseng and bacopa. Whilst research is lacking in some respects for all of these extracts, they are all safe and well tolerated, and none are associated with significant negative effects on any parameter. Alkaloids, as the archetypal plant defence compounds, often have intense effects on neurotransmission in herbivores and human consumers. However, their potency, toxicity and legal status mean that only nicotine and caffeine currently have any obvious potential role in cognitive enhancement in a sporting context. However, nicotine is addictive, and the contribution of habituation and withdrawal effects, and therefore dosage patterns, for both compounds, need to be taken into consideration. Some interesting data are emerging with regard to potential additive effects and synergies when phytochemicals and low doses of caffeine are co-consumed, and phytochemical or nutrient-rich extracts containing low doses of caffeine might be a rational approach for enhancing cognitive function.

Whilst several of the phytochemicals reviewed above would seem to have potential utility in terms of improved cognitive function or psychological state in healthy adults, the current lack of direct evidence collected in a sporting context means that no firm recommendations are possible at this time. The efficacy of phytochemicals, in particular the benign phenolics and terpenes reviewed above, and the potential for positive, or indeed negative, interactive effects between their psychoactive properties and the physical and psychological arousal intrinsic to most sports deserves more research attention.
